# Adapting an In-Home Randomized Intervention Trial Protocol for COVID-19 Precautions

**DOI:** 10.3390/ijerph20031987

**Published:** 2023-01-21

**Authors:** Shir Lerman Ginzburg, Teresa Vazquez-Dodero, Chermaine Mason, Neelakshi Hudda, Leigh Meunier, Linda Sprague Martínez, Misha Eliasziw, Doug Brugge

**Affiliations:** 1 Department of Public Health, School of Arts and Sciences, MCPHS University, Boston, MA 02115, USA; 2 Department of Public Health Sciences, University of Connecticut Health Center, Farmington, CT 06030, USA; 3 Department of Civil and Environmental Engineering, Tufts University, Medford, MA 02476, USA; 4 Macro Department, Boston University School of Social Work, Boston, MA 02215, USA; 5 Department of Public Health and Community Medicine, Tufts University, Boston, MA 02111, USA

**Keywords:** clinical trials, COVID-19, environmental health, methodologies, protocol revision

## Abstract

Background: The COVID-19 pandemic has significantly impacted the status of clinical trials in the United States, requiring researchers to reconsider their approach to research studies. In light of this, we discuss the changes we made to the protocol of the Home Air Filtration for Traffic-Related Air Pollution (HAFTRAP) study, a randomized crossover trial of air filtration in homes next to a major highway. The senior authors designed the trial prior to the pandemic and included in-person data collection in participants’ homes. Because of the pandemic, we delayed the start of our trial in order to revise our study protocol to ensure the health and well-being of participants and staff during home visits. To our knowledge, there have been few reports of attempts to continue in-home research during the pandemic. Methods: When pandemic-related protective measures were imposed in March 2020, we were close to launching our trial. Instead, we postponed recruitment, set a new goal of starting in September 2020, and spent the summer of 2020 revising our protocol by developing increased safety precautions. We reviewed alternative approaches to installing portable air filtration units in study participants’ homes, in order to reduce or eliminate entry into homes. We also developed a COVID-19 safety plan that covered precautionary measures taken to protect both field team staff and study participants. Results: Our primary approach was to minimize contact with participants when collecting the following measures in their homes: (1) placing portable air filtration units; (2) conducting indoor air quality monitoring; (3) obtaining blood samples and blood pressure measurements; and (4) administering screening, consent, and follow-up questionnaires that coincided with collection of biological measures. Adapting our public health trial resulted in delays, but also helped ensure ethical and safe research practices. Perceived risk of COVID-19 infection appeared to have been the primary factor for an individual in deciding whether or not to participate in our trial, particularly at the beginning of the pandemic, when less was known about COVID-19. Conclusions: We needed to be flexible, creative, and calm when collaborating with community members, the IRB, and the universities, while repeatedly adjusting to changing guidelines as we determined what worked and what did not for in-home data collection. We learned that high-quality air monitoring data could be collected with minimal in-person contact and without compromising the integrity of the trial. Furthermore, we were able to collect blood pressure and phlebotomy data with minimal risk to the participant.

## 1. Introduction

The COVID-19 pandemic has significantly affected the status of clinical trials in the United States, especially those that were in the early stages or recruiting when the World Health Organization declared a pandemic in March 2020. The pandemic delayed recruitment of participants, data collection, and supervised treatment, and also affected the capacity of hospitals and laboratories to conduct research [1]. Unlike most conventional clinical trials, our study involved entering participants’ homes on four separate occasions, as well as prolonged interaction with participants, to collect data. To our knowledge, there have been no reports of how to continue conducting in-home research during the pandemic. Comparing study protocols before, during, and as restrictions eased might provide valuable insights into effective adaptations to such circumstances.

As the pandemic progressed, researchers needed to think about how to modify clinical trial protocols to a world impacted by COVID-19 [2]. Some studies utilized research facilities closer to the participants’ homes and used video communication for study components that did not require in-person interactions [3]. Other studies delayed study components that were not critical to the proposal’s main aims with the hope of eventual implementation at a safer time. Laboratories had to quickly adapt to testing for COVID-19 infections [4,5,6].

When and how to continue or recommence clinical trials raised questions about the conduct of research with respect to the health and safety of both study participants and project staff. Vissers and colleagues (2021) drew on the Belmont Report to recommend adhering to bioethical standards with the reopening of clinical trials, including: (1) autonomy, by which investigators have a responsibility to inform study participants about any known or potential changes in the benefits, risks, and conduct of the trial as a result of the developing pandemic, so that participants could make informed decisions; (2) beneficence and nonmaleficence, which concerns the potential or perceived benefits and risks to both the participant and staff; and (3) justice, which addresses the fair distribution of scarce resources, the participant’s rights, and respect toward morally acceptable laws [7].

In this paper, we discuss our approach to initiating our trial toward the end of the first year of the COVID-19 pandemic and compare it to the second year of participant recruitment, when serious illnesses caused by COVID-19 had declined. Although our trial was designed at the outset to collect data in participants’ homes, this approach had to be reevaluated when the pandemic shut down many research studies. We present our reflections as a case study of protocol adaptations, with lessons drawn from our experience.

## 2. Materials and Methods

Our public health trial, Home Air Filtration for Traffic-Related Air Pollution (HAFTRAP), is a randomized crossover trial of in-home High Efficiency Particulate Arrestance (HEPA) filtration on cardiovascular health among residents who live near a highway in the Greater Boston Area. The basis for the trial is that elevated particulate pollution next to highways increases cardiovascular disease risk [8,9] and air filtration units have been shown to improve the indoor air quality of homes, which in turn may reduce cardiovascular disease risk.

We designed our NIH-funded trial a couple of years before the start of the pandemic. The field protocol included in-home walkthrough surveys, as well as blood pressure measurements and the collection of blood samples and questionnaires in participants’ homes at four time points over a three-month period. The intervention was the installation of portable HEPA air filtration units in the bedroom and living room of homes of each study participant during the cooler months of the year (September through March). Home walkthroughs were designed to assess physical characteristics of the participant’s home (such as types of windows and doors). For a subset of participants, we sought to monitor residential air-quality and/or personal-level air quality. This work was envisioned to involve direct, in-home interaction with study participants.

When pandemic-related protective measures, including physical distancing, were imposed in March 2020, we were close to launching the trial. Instead, we postponed recruitment and set a new goal of starting in September 2020 in order to revise our trial protocol in a way that would allow for safe data collection while limiting the time spent in participants’ homes. As this study required that measures be collected in participants’ homes, we spent the summer of 2020 developing the increased safety precautions that we needed to ensure the health and well-being of both study staff and participants without compromising data quality. We considered changes to study materials, Institutional Review Board (IRB) applications, eligibility screening, informed consent, HIPAA forms, participant questionnaires, and biological data (phlebotomy and anthropometry) collection. We also reviewed alternative approaches to installing the portable air filtration units in study participants’ homes to reduce or eliminate entry into homes.

## 3. Procedure

We revised our protocol, data collection forms, and procedures to comply with federal, state, and university safety recommendations and mandates as they emerged, and as our understanding of the risk of infection grew. We also developed and submitted a COVID-19 safety plan that covered precautionary measures taken to protect both the field team staff and the study participants. These measures included wearing face masks at all times while inside the participants’ homes, sanitizing equipment before and after contact with staff and participants, and minimizing in-person contact by conducting the screening and informed consent/HIPAA questionnaires virtually. We obtained approval for our plan from UConn Health, the lead university for this study.

The changes we made evolved over time in response to changes in local and federal rules and guidance, as well as based on what we learned from ongoing experience. In March 2020, UConn Health restricted out-of-state travel, which blocked our postdoctoral fellow from traveling from Connecticut to Massachusetts to conduct in-person screening and baseline questionnaires with participants. In March 2020, our principal investigator consulted with our Program Officer at the National Institutes of Health about our plans and received assurance that our modified plan was acceptable. Our overall approach was to minimize contact with participants when placing portable air filtration units in their homes, conducting indoor air quality monitoring, obtaining blood samples and blood pressure measurements, and administering screening, consent, and follow-up questionnaires. Table 1 provides a timeline of our trial, both before and during the pandemic, in order to highlight the delays in starting recruitment due to COVID-19.

## 4. Results

Changes to the trial were made in several areas, including revisions to the protocol, recruitment strategies, and data collection.

### 4.1. Revisions to the Trial Protocol

Obtaining IRB approval for the revised protocol significantly delayed our start date. Some of the modifications required two or three rounds of IRB submissions. We also obtained a waiver of signed consent from the IRB so that the participants would not need to sign physical copies of the informed consent and HIPAA forms. Instead, the postdoctoral fellow met with each participant either over Zoom or the phone and read the informed consent and HIPAA forms out loud. Prospective participants had the opportunity to ask questions at any point in this process. If the participant had an email address, the postdoctoral fellow emailed him or her a copy of the forms signed by the postdoc. If the participant did not have an email address, then the postdoctoral fellow emailed the forms to the project manager to print and give to the participant at the first home visit.

We also significantly revised the protocol to institute safety measures for study staff and participants. We identified questions and measures that were problematic when administered remotely, notably the neurocognitive exam, which required the participant to trace numbers and letters on a surface. We considered a verbal-only version of the measure, but decided against it because the data quality was lower, although we did give the participant the option to take this form remotely. However, none of the participants decided to complete this form remotely. We also added a question about COVID-19 vaccination status to the eligibility screener and added questions on COVID-19 exposure to the baseline and follow-up questionnaires to capture symptoms, diagnosis, and problems with wearing a face mask.

### 4.2. Recruitment

Because the air pollution exposure of interest as seasonal (i.e., there are higher concentrations of pollution during colder weather), we had pushed back our recruitment start date from spring 2020 to fall 2020. We found that recruitment was slow, primarily due to concerns of prospective participants about COVID-19 infection and having strangers enter their homes. This was apparent because some prospective participants informed our project manager of their discomfort participating in a study during the pandemic that required close contact with strangers. In our experience, older participants, who were at increased risk of COVID-19 compared to younger adults, were more reluctant to enroll in the trial. However, most of the individuals who enrolled remained for the duration of the trial. Once enrolled, none dropped out due to fears about COVID-19 exposure.

A centerpiece of our recruitment strategy was collaboration with teenagers in the Liaison Interpreters Program of Somerville (LIPS), sponsored by The Welcome Project, one of our community partners. The LIPS teenagers served as participant recruiters. Our approach to training the youth had to evolve to address the COVID-19 pandemic, as we had an obligation to ensure the safety of the teens as well as the community. Before COVID-19 was upgraded to a pandemic, we envisioned recruitment with the LIPS teens to unfold in two ways: first, through hanging flyers at local businesses along streets proximate to the study area, and second, through direct knocking on the doors of residential homes in the study area. For training and recruitment, our project manager met virtually with several second-year LIPS teenagers to strengthen the teens’ understanding of the HAFTRAP study, community-based research, and ethical considerations (e.g., consent; confidentiality). All of these interactions were conducted virtually.

Over the summer of 2020, the HAFTRAP team and Welcome Project staff drafted a COVID-19 safety plan for the teens, updated the recruitment flyer, and created a door hanger in order to reach residents at their homes while avoiding face-to-face COVID-19 transmission risks. As fall 2020 approached, the youth and project manager conducted recruitment by distributing flyers and placing door hangers on homes in the study area. We ensured that the youth wore masks and practiced physical distancing.

After encountering slow recruitment, we decided to modify the inclusion and exclusion criteria. Specifically, we reduced the lower age cut-off from 40 to 30 years of age. Our rationale was that elevated blood pressure, our primary outcome, begins to develop under the age of 40. We also revised our mechanical air handling system exclusion criterion so that people with air conditioning units could participate (although having forced air through vents remained an exclusion).

The PI’s postdoctoral fellow conducted the eligibility screening process virtually with potential participants who were referred to her by the project manager. We screened our first study participants in September 2020, six months after the pandemic started in the United States. We began the first interventions in November 2020.

For eligible participants, the postdoctoral fellow read the informed consent and HIPAA forms to the participant over the phone or on Zoom. Once consent had been obtained, the postdoctoral fellow administered the baseline questionnaire, which asked about demographic information, health, and the home environment. Subsequently, she provided the project manager with the participants’ contact information so she could schedule the installation of the filtration unit, as well as schedule the initial home visits for collection of biological samples.

### 4.3. Data Collection

The project manager and the phlebotomist went to the participants’ homes together in order to decrease the number of home visits. They asked participants if they were comfortable with the precautions that we took in order to maintain open and honest communication, which we believe helped to mitigate participants’ concern. Field team staff always wore masks and gloves and took off their shoes when entering participants’ homes.

Our initial plan was to measure blood pressure and collect blood samples in apartment building hallways or on porches to avoid entering participants’ homes. However, because of cold temperatures and a lack of enclosed spaces outside most apartments, this was not possible. In addition to participant comfort, we were concerned that temperatures below those maintained in living spaces could affect blood pressure measurements [10,11,12].

Accordingly, we decided to enter participants’ homes to measure blood pressure and collect blood samples, a practice which the phlebotomist and her team were already following in their clinical work. To minimize risk, we set up the phlebotomy table by a door or window to allow for better air circulation, and we prepared all of the equipment prior to inviting the participant to sit down for the blood draw in order to maintain as much physical distancing as possible.

We installed the air quality monitoring equipment on a cart that was rolled into the participants’ homes, and we programmed the equipment to record data without needing any further interaction other than being plugged in by participants. Outdoor monitoring plans remained unchanged. One consequence of this approach is that we did not have access to indoor instruments for quality control checks. We cancelled the home walkthroughs envisioned in the original study protocol because we deemed that this information would not change and could be filled in at a later date.

Study staff dropped off the portable air filtration units by carrying them up to the participant’s front door, as well as up the stairs if the residence was not on ground floor. Participants would then take the units into their homes and install them while on the phone with study staff. Study staff retrieved the units prior to the second intervention sessions (start of the third month) in order to switch them from sham to HEPA filters or vice versa. We purchased a powered air-purifying respirator for our project manager to wear while changing the filters. This was prompted partly by the possibility of a slight risk of viable COVID-19 particles in the air filtration units, but also because the dust they trapped could contain allergens or other contaminants. Table 2 highlights the changes we made to our trial protocol.

Because our interventions were only implemented during the cool to cold months, we took the summer of 2021 to reassess whether we needed to make any changes to our study materials. While we did not make any content changes to the study materials, we did have all study materials translated into Spanish so we could recruit a wider range of individuals. The Spanish-level documents were approved by the University of Connecticut Health Center IRB in the summer of 2021. The postdoctoral fellow and the project manager both speak fluent Spanish and began recruiting and enrolling Spanish-speaking participants in fall of 2021. We also hired an additional recruiter to help the project manager with outreach. With the introduction of the COVID-19 vaccinations and boosters throughout 2021, we found that recruitment and enrollment efforts improved (Table 3).

Of the 29 individuals who were screened for eligibility between September 2020 and March 2021, 16 (55.2%) were enrolled in the trial. In contrast, a year later (September 2021 through March 2022), enrollment nearly tripled to 42 participants, and nearly all 46 individuals who were screened were enrolled into the trial. The 13 individuals in 2020–2021 were screened out for one or more of the following reasons: five had a mechanical air handling system in their home; two had sources of smoke in their living space every day; three had cancer requiring treatment; and seven were unreachable prior to signing consent. In contrast, the four individuals who screened out in 2021–2022 were simply unreachable.

Table 4 summarizes and compares the characteristics of the 16 participants who were enrolled in 2020–2021 to the 42 participants who were enrolled a year later, in 2021–2022. No significant differences were observed between these two cohorts of participants, although more Hispanic participants with lower annual household incomes were enrolled during 2021–2022 because all study materials were now available in Spanish.

## 5. Expected Results

The most important lesson learned while revising our study protocol was that we had to be flexible and creative in collaborating with community members, the IRB, and the universities while repeatedly adjusting to changing guidelines as we determined what worked and what did not for in-home data collection. We were able to overcome obstacles while still maintaining high safety standards for both participants and study staff, although this forced us to push back our timeline and recruitment ended up being below our target. We also learned that high-quality air monitoring data could be collected with minimal in-person contact and without compromising the study’s aims. Furthermore, we were able to collect blood pressure and phlebotomy data with minimal risk to the participant.

Our project manager found that interacting with participants in their homes required an extra level of calmness, particularly during the chaotic morning rush. As schools were either not in session or held virtually during our fieldwork, we had to be flexible to accommodate both participants and staff with children. However, because participants were spending more time at home, there were some advantages to engaging with those willing to participate, as they might have otherwise been too busy with work and other obligations to participate in the study.

Changes were initially difficult to implement due to changing federal and local recommendations and mandates, uncertainty about how long the pandemic would last and what precautions were most effective, and our own learning curve as we adjusted to running a research study that was originally designed to require close physical contact and entering participants’ homes. While changes were initially made through ongoing experience, we feel that we ultimately arrived at a reasonable approach that was effective at protecting participant and staff safety while allowing us to implement an in-home intervention during the COVID-19 pandemic. Our experience highlights the importance of team collaboration, critical thinking, and creativity in implementing research methods to ensure safety and health while maintaining research integrity.

### Lessons Learned

This paper discussed measures taken at the onset of the COVID-19 pandemic to adapt the protocol for our in-home public health intervention trial to be compliant with federal and local COVID-19 safety procedures, as well as to maintain ethical and safe research practices. To our knowledge, there are no reports of sustained research during the pandemic that required contact in the homes of study participants. Despite challenges that resulted in delays, we succeeded in starting and sustaining recruitment of participants into our intervention study by adhering to the Belmont Report’s ethical principles of Beneficence, Respect for Persons, and Justice. Moreover, the characteristics of the participants whom we recruited during the pandemic did not differ from those recruited a year later, when the pandemic was subsiding.

Abiding by the Respect for Persons principle, we acknowledged that study participants were autonomous individuals with the capacity to make their own decisions, particularly regarding health-related matters. We were transparent with study participants about COVID-19 risk and our protective measures, and were also flexible with both scheduling and with the participants’ comfort levels in having strangers in their homes during a global pandemic.

Adhering to the Beneficence principle, we conducted the screening and informed consent/HIPAA processes over the phone in order to eliminate face-to-face contact for this aspect of data collection. We also minimized contact during home visits by the project manager and phlebotomist, both of whom always wore face masks when in contact with participants, indoors or outdoors.

Although choice of neighborhoods preceded COVID-19 by years, we think there is an aspect to the Justice principle that applies to changes in our study. As the study neighborhoods were already at elevated risk of COVID-19, we felt that we needed to be particularly cognizant that they were a vulnerable population. Actively collaborating with local community organizations to assist in recruiting and engaging participants helped us to tailor our approach to the specific context of our study area.

Despite our precautions, the socially-perceived risk of COVID-19 appeared to be a significant factor in peoples’ decisions regarding whether or not to participate in the study. Nevertheless, we were still able to recruit about one-third of the number of participants we originally sought to enroll in the first year of the study, which was better than suspending recruitment entirely. Furthermore, recruitment levels improved once vaccinations became widely available, particularly as study staff were also vaccinated, which added to the participants’ comfort with permitting staff into their homes.

## 6. Conclusions

COVID-19 had a major impact on research, including our own, as study participants were uncomfortable with allowing strangers into their homes. As such, we had to revise our study protocol and other materials to adhere to federal and state safety guidelines. Such changes included minimizing in-person contact with participants and always wearing masks. By adhering to strict safety measures and ethical principles about prioritizing the safety of participants and study staff, we were able to recruit more participants than expected. Revising study materials during a global pandemic requires creativity, critical thinking, and team cooperation.

## Figures and Tables

**Table 1:** Study timeline.

Action	Date
Initial IRB approval for trial	January 2020
Pandemic declared; lockdown commences in the United States	March 2020
Decision to postpone start of field work	March 2020
Major revisions made to trial protocol	April–July 2020
IRB approval or revised protocol	July 2020
Change in project manager	June–October 2020
Participant recruitment begins, Year 1	September 2020
Participant screening, Year 1	September 2020–March 2021
Start of intervention, Year 1	November 2020
End of intervention, Year 1	July 2021
Participant recruitment begins, Year 2	September 2021
Participant screening, Year 2	September 2021–March 2022
Start of intervention, Year 2	September 2021
End of intervention, Year 2	June 2022

**Table 2:** Changes made to HAFTRAP after the COVID-19 restrictions in March 2020.

	Intended Pre-COVID-19 Research Plans	Revisions Made during COVID-19 Pandemic	Belmont Report Principles
Personal protection of field staff (project manager and phlebotomist)	Only protection for blood-borne pathogens was envisioned	Purchase of a respirator for the project manager to use while cleaning out used air filters. Added to the protocol that staff were to wear face masks at all times when interacting with participants. Blood pressure measurements and blood draws were initially attempted outdoors or in hallways.	Beneficence
Eligibility/screener	Planned to conduct in person Exclusion of homes with mechanical air handling systems	Revised mechanical air handling system exclusion criterion so that people with air conditioning units could participate (although forced air through vents was still an exclusion). We revised this exclusion criterion in order to increase recruitment. Added questions on COVID-19 exposure o Symptomology o Diagnosis o Problems with wearing a face mask Conducted the screening virtually.	Beneficence
Informed consent form	To be conducted in person Signed informed consent by both study staff and participant	Conducted over the telephone or teleconferencing. Obtained a waiver of signed consent from the IRB so that participants would not need to sign the form, although the project manager provided them with a physical copy of the form.	Beneficence
HIPAA authorization	Signed by both study staff and participant	Conducted virtually. Obtained a waiver so that participants would not need to sign the form, although the project manager provided them with a physical copy.	Beneficence
Air filtration equipment installation	Study staff planned to enter the participants’ homes to install the air filters and connect them to an electricity metering device that recorded filter use	Field team staff dropped off filters (hooked into electricity metering devices) at the participants’ front doors for participants to plug in and start themselves. Participants told the project manager where they placed the air filters, either verbally or via text; sometimes the project manager also observed their placement when she entered their homes.	Respect for persons Beneficence
Air quality monitoring	Researchers planned to enter the participants’ homes to install the air quality monitoring equipment with a pump housed at a location (such as a closet) that limited noise Researchers planned to access the outdoor property to install the air quality monitoring equipment in weatherproof boxes to monitor outdoor concentrations Researchers would have access to the sites (indoor and outdoor) to conduct quality control checks on instruments midway through the sampling period	Researchers installed the air quality monitoring equipment with quieter pumps on a cart that could be rolled into the home, and started/programmed the equipment to record data without needing any further interaction with the equipment other than simple plug-in. Outdoor monitoring plans remained unchanged. Researchers did not have access to indoor sites to conduct quality control checks.	Respect for persons Beneficence
Personal exposure monitoring (PEM)	Researchers would hand off the PEM case to participants and explain the operation in a hands-on manner; researchers would troubleshoot in-person if required	Researchers handed off the PEM case to participants while practicing social distancing, with written instructions for participants. Troubleshooting was performed via phone if necessary.	Beneficence
Home walkthrough	Home walkthroughs were to be conducted by air monitoring staff	On hold until the pandemic eases. After we started entering the participants’ homes to collect biospecimens, we decided to keep this measure on hold in order to minimize the amount of contact with participants.	Beneficence

**Table 3:** Comparison of recruitment and enrollment efforts between winter 2020/spring 2021 and winter 2021/spring 2022.

Activity	Winter 2020/Spring 2021 (September 2020–March 2021)	Winter 2021/Spring 2022 (September 2021–March 2022)
Recruitment	29 participants screened 16 participants enrolled from 12 households 55.2% enrollment rate	46 participants screened 42 participants enrolled from 29 households 91.3% enrollment rate
Home walkthroughs	None	26
Personal monitoring	None	5
Air monitoring	None	18

**Table 4:** Comparison of characteristics of HAFTRAP participants between winter 2020/spring 2021 and winter 2021/spring 2022.

	Winter 2020/Spring 2021 (September 2020–March 2021) N = 16	Winter 2021/Spring 2022 (September 2021–March 2022) N = 42
Age in years, mean (sd)	44.2 (10.5)	44.2 (12.6)
Gender, *n* (%)		
Male	5 (31.3)	13 (31.0)
Female	11 (68.7)	29 (69.0)
Ethnicity/race, *n* (%)		
Hispanic	3 (18.7)	11 (26.2)
Non-Hispanic White	10 (62.5)	26 (61.9)
Non-Hispanic Black	2 (12.5)	2 (4.8)
Non-Hispanic Asian	0 (0.0)	3 (7.1)
Non-Hispanic Other	1 (6.3)	0 (0.0)
Education, *n* (%)		
<5th grade	0 (0.0)	0 (0.0)
6th to 12th grade	3 (18.7)	7 (16.7)
Some college	5 (31.3)	5 (11.9)
College or university degree	8 (50.0)	30 (71.4)
Employment status, *n* (%)		
Unemployed	4 (25.0)	11 (26.2)
Working part-time	4 (25.0)	6 (14.3)
Working full-time	8 (50.0)	25 (59.5)
Annual household income, *n* (%)		
<$48,000	0 (0.0)	4 (9.5)
$48,000 or more	9 (56.3)	27 (64.3)
Declined to answer	7 (43.7)	11 (26.2)
Central blood pressure, mean (sd)		
Systolic pressure (mmHg)	113.4 (14.0)	113.4 (12.6)
Diastolic pressure (mmHg)	80.7 (11.2)	80.5 (9.6)
Pulse pressure (mmHg)	32.7 (5.7)	32.8 (7.9)
Mean pressure (mmHg)	95.1 (12.8)	95.4 (10.2)

## Data Availability

The datasets generated and/or analyzed during the current study are not publicly available due ongoing data-collection, but are available from the senior author upon reasonable request. To request data and materials, please contact: Doug Brugge, Department of Public Health Sciences, University of Connecticut Health Center, 263 Farmington Ave, MC 6325, Farmington, CT 06030-6325, 860-679-8814, brugge@uchc.edu.

## Abbreviations

COVID-19	Coronavirus Disease
HAFTRAP	Home Air Filtration for Traffic-Related Air Pollution study
HEPA	High Efficiency Particulate Arrestance air filters
HIPAA	Health Insurance Portability and Accountability Act
IRB	Institutional Review Board
LIPS	Liaison Interpreters Program of Somerville
NIH	National Institutes of Health
PI	Principal Investigator
TWP	The Welcome Project
